# Metabolic Signatures in Response to Abscisic Acid (ABA) Treatment in *Brassica napus* Guard Cells Revealed by Metabolomics

**DOI:** 10.1038/s41598-017-13166-w

**Published:** 2017-10-09

**Authors:** Mengmeng Zhu, Sarah M. Assmann

**Affiliations:** Department of Biology, Pennsylvania State University, Pennsylvania 16802 University Park, USA

## Abstract

Drought can severely damage crops, resulting in major yield losses. During drought, vascular land plants conserve water via stomatal closure. Each stomate is bordered by a pair of guard cells that shrink in response to drought and the associated hormone abscisic acid (ABA). The activation of complex intracellular signaling networks underlies these responses. Therefore, analysis of guard cell metabolites is fundamental for elucidation of guard cell signaling pathways. *Brassica napus* is an important oilseed crop for human consumption and biodiesel production. Here, non-targeted metabolomics utilizing gas chromatography mass spectrometry (GC-MS/MS) and liquid chromatography mass spectrometry (LC-MS/MS) were employed for the first time to identify metabolic signatures in response to ABA in *B. napus* guard cell protoplasts. Metabolome profiling identified 390 distinct metabolites in *B. napus* guard cells, falling into diverse classes. Of these, 77 metabolites, comprising both primary and secondary metabolites were found to be significantly ABA responsive, including carbohydrates, fatty acids, glucosinolates, and flavonoids. Selected secondary metabolites, sinigrin, quercetin, campesterol, and sitosterol, were confirmed to regulate stomatal closure in *Arabidopsis thaliana*, *B. napus* or both species. Information derived from metabolite datasets can provide a blueprint for improvement of water use efficiency and drought tolerance in crops.

## Introduction

Plants are sessile organisms that are continuously subjected during their lifecycles to a spectrum of environmental signals and stimuli, including both abiotic factors such as availability of water, light, and nutrients, and biotic factors such as interactions with both beneficial and pathogenic organisms. Accordingly, plants have evolved a suite of molecular mechanisms for external signal perception and transduction, facilitating acclimation to diverse environmental conditions^1^. Water deficit is one of the major abiotic stresses causing severe losses in crop production^2^. Drought triggers the biosynthesis, accumulation, and redistribution of abscisic acid (ABA), which promotes stomatal closure, inhibits stomatal opening, and thereby reduces water loss^3–7^. The pivotal role of stomata in ABA interactions during drought stress has resulted in extensive efforts to elucidate the ABA signaling pathways of guard cells, which border and regulate stomatal apertures. Genetic screens and recent systems biology studies have revealed many signaling events and molecular components that participate in ABA signaling^8–15^.

Knowledge of ABA signaling in guard cells has been largely derived from the model plant *Arabidopsis thaliana*, which has limited economic value. *Brassica napus*, also known as oilseed rape or rape, is one of the largest commercial sources of vegetable oil. *B. napus* is grown worldwide for both human consumption and biodiesel production. *B. napus* is susceptible to drought stress, which can cause severe reduction in oilseed production^16^. An improved understanding of molecular responses to ABA in *B. napus* guard cells will inform genetic engineering and breeding approaches to enhance drought tolerance in crops.

Large scale *B. napus* guard cell protoplast isolation from *B. napus* leaves can be conducted with high purity and yield, which provides optimal material for –omics analyses on this single cell type^17–19^. Using an iTRAQ (isobaric tag for relative and absolute quantitation)-based comparative proteomics approach, 66 and 38 proteins were found to be significantly induced and suppressed by ABA in *B. napus* guard cells, respectively. These ABA responsive proteins participate in photosynthesis, metabolism, energy, protein synthesis, stress/defense (antioxidant system and glucosinolate-myrosinase system), membrane and transport processes, and protein folding/transport and degradation^10^. Recently, 65 thiol-based redox responsive proteins were identified from ABA-treated *B. napus* guard cells, which highlights redox switches as important regulatory mechanisms in ABA signal transduction in guard cells^14^.

Metabolites are direct physiological signatures and are highly correlated with phenotypes^20^; thus, study of cellular metabolomics is also indispensable for complete understanding of stress responses. Stress responsive metabolomes have been investigated in cell culture and in whole plants or whole organs, but rarely in single cell types^21,22^. One landmark application of metabolomics to study the stress regulated metabolome at the level of the single cell type was an investigation of the ABA responsive metabolic changes in guard cell protoplasts from *A. thaliana* wild type and heterotrimeric G-protein α subunit mutant, *gpa1*, using targeted metabolomics with multiple reaction monitoring (MRM)^12^. In targeted metabolomics, paired mass/charge (*m/z*) ratios of the precursor ion and a selected daughter ion along with the chromatographic retention time, as acquired from analysis of authentic compounds (standards), are employed to identify a metabolite in experimental samples. Eighty-five signaling-related metabolites in *A. thaliana* guard cells were detected and quantified. The abundance of nearly half of these metabolites (41 out of 85) in wild type guard cells was significantly changed after ABA treatment. Interaction with other hormones, particularly indole-3-acetic acid (IAA), in ABA modulated stomatal movement was revealed, validating phytohormone crosstalk^12^. These targeted MRM-based profiles of the *A. thaliana* guard cell metabolome provided the first example of investigating dynamic metabolome changes of a single-cell-type in plants.

Plant metabolomes are highly diverse and have been recognized for their nutritional and medicinal value for centuries^23^. There are an estimated ~200,000 metabolites produced by the plant kingdom^24^. To date, however, only ~100 metabolites have been identified in guard cell protoplasts or implicated in guard cell functions^18^. The majority of these metabolites were identified in the targeted metabolomics study of Jin *et al*.^12^, while others were identified in focused studies on a specific metabolite or metabolic pathway. Recently, using guard cell enriched epidermal peels prepared from *B. napus* leaves, a material relatively easier to obtain, the guard cell metabolite inventory has been expanded to a few hundred metabolites, based on discovery from both targeted and non-targeted metabolomics platforms^25,26^.

Non-targeted metabolomics provides a complementary approach to targeted metabolomics, with the aim to acquire not only the mass/charge ratio but also the tandem mass spectra of all detected precursor molecules^20^. Such information facilitates elucidation of the chemical structure of each molecule. Instead of selective detection of a pre-defined metabolite group, non-targeted metabolomics provides global information on the metabolome. To improve our knowledge of the functional guard cell metabolome, here we employed non-targeted metabolomics workflows utilizing two complementary platforms, i.e., gas chromatography (GC)-mass spectrometry (MS) and liquid chromatography (LC)-MS to profile the *B. napus* guard cell metabolome and its modulation by ABA, resulting in a profile of 390 non-redundant metabolites, 77 of which were ABA responsive. Based on these results, several secondary metabolites were chosen for targeted study and were found to show either antagonistic or additive effects on ABA-induced stomatal closure. Information derived from metabolite datasets will improve our knowledge of ABA signaling in guard cells.

## Results

### Physiological stomatal response to ABA in *B. napus*

**Figure 1:**
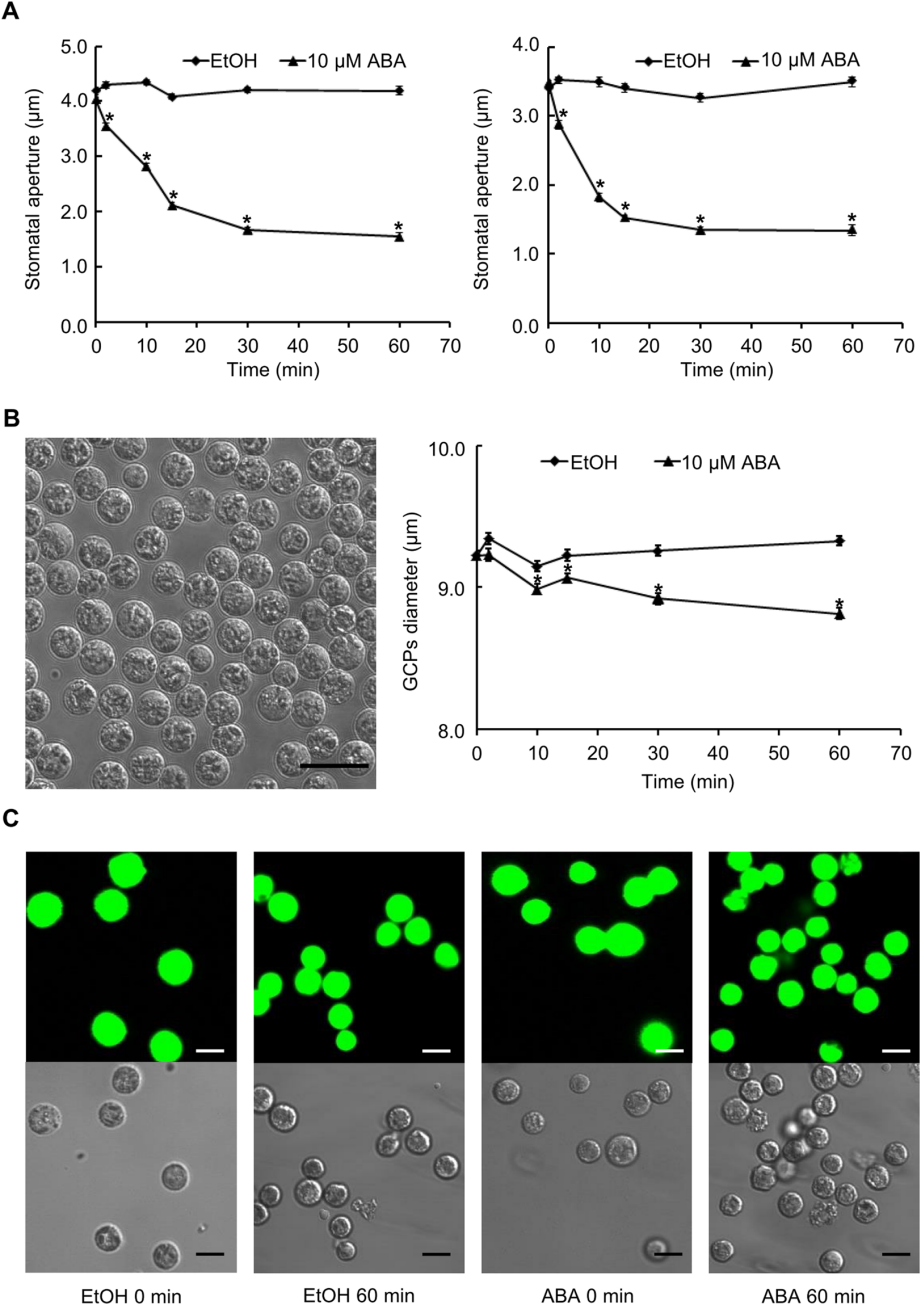
Responses to ABA in *B. napus* leaves, epidermal peels, and guard cell protoplasts. (**A**) ABA (10 µM) induces stomatal closure in both leaf pieces (left panel) and epidermal peels (right panel) of *B. napus* line DH12075. Data are means ± standard errors of 3 independent replicates with 100 ± 5 stomata measured for each sample. (**B**) ABA-induced shrinkage of *B. napus* GCPs. Representative image (left); scale bar indicates 25 µm. Data (right) are means ± standard errors of 4 independent replicates with 100 ± 5 GCPs measured for each sample. (**C**) *B. napus* GCPs are viable following ABA or ethanol *(*solvent control) treatment. Samples before treatment (0 min) and after treatment (ethanol (EtOH) 60 min and ABA 60 min) were FDA stained to assess cell viability. Scale bars indicate 10 µm. Asterisks in A and B indicate that ABA treatment differed significantly from the EtOH solvent control (Student’s *t* test; *p* < 0.05).

ABA regulated stomatal movement has been observed in a wide range of plant species, including *A. thaliana* and *B. napus* ^10,12,27,28^. Here we first confirmed that 10 µM ABA, a concentration typically used in assays of stomatal responses^29–31^, is sufficient to induce stomatal closure in both leaf pieces (Fig. 1A) and epidermal peels (Fig. 1A) of *B. napus* line DH12075. ABA-induced stomatal closure was observed within 2 min and closure was complete within 30 min of treatment in both materials (Fig. 1A). These results indicate the effectiveness of the ABA concentration used for our subsequent metabolomics analyses on *B. napus* guard cell protoplasts (GCPs). We also confirmed that the solvent for ABA application, ethanol, had no effect on stomatal apertures (Fig. 1A).

Although epidermal peels have been used for guard cell related –omics studies^11,25,26,32^, our metabolome analyses were performed on guard cell protoplasts, rather than on epidermal peels, in order to exclude metabolites arising from pavement cells and the cuticle. The viability of isolated GCPs (Fig. 1B) before and after ABA or ethanol treatment was confirmed by fluorescein diacetate (FDA) staining (Fig. 1C). ABA responsiveness of the GCPs was evaluated by measuring protoplast diameters over a time course of ABA treatment (Fig. 1B). GCP shrinkage was observed when GCPs were treated with 10 µM ABA as compared to the ethanol (solvent) control (Fig. 1B and C). Together, these results confirm that viability and ABA responsiveness were maintained in *B. napus* GCPs after protoplasting and treatment.

### Metabolome profiling of *B. napus* guard cells

**Figure 2:**
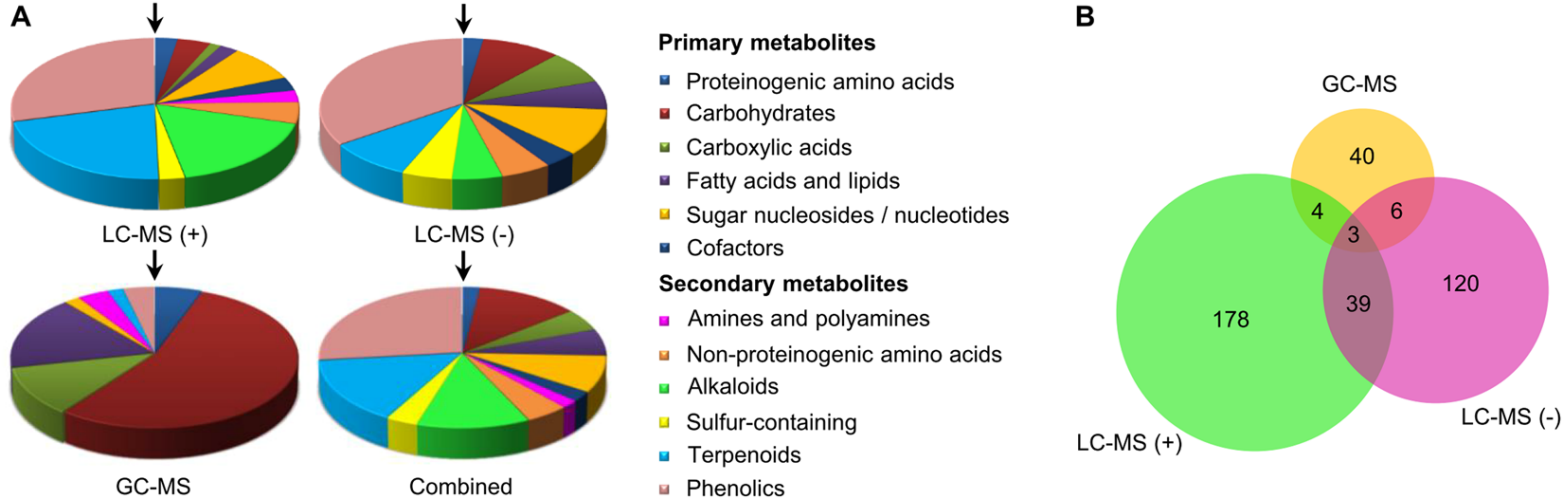
Metabolomic profiling using complementary platforms resulted in identification of 390 non-redundant metabolites in *B. napus* GCPs. (**A**) Classification of metabolites identified from each platform, mainly based on structural characteristics^101^. The metabolite categories are listed in the figure caption in the clock-wise order in which they appear in the figure, starting with “Proteinogenic amino acids” at the 12 o’clock position (arrow) in all four pie charts. No metabolites in the category of “Amines and polyamines” were identified by the LC-MS (−) platform. No metabolites were identified by GC-MS in the categories of “Cofactors”, “Non-proteinogenic amino acids”, “Alkaloids”, or “Sulfur-containing”. (**B**) Venn diagram showing the number of metabolites identified from each platform.

Two major objectives of this study were metabolome profiling and identification of ABA responsive metabolites in *B. napus* guard cells. We prepared a total of 226 million *B. napus* GCPs, obtained in ~50 GCP isolations from ~1500 g of *B. napus* leaves (fresh weight), for our metabolomics analyses. After protoplasting, *B. napus* GCPs were left untreated or exposed to a time course of ABA treatment (see next section). Pre-separation by gas chromatography (GC) or liquid chromatography (LC) coupled with tandem mass spectrometry are robust methods to generate fragmentation patterns that can yield definitive metabolite identification. Here we first employed both GC-MS/MS and LC-MS/MS to explore the *B. napus* guard cell metabolome. Five replicates, each with 4–4.5 million untreated GCPs, were prepared and analyzed on the two platforms. For our GC-MS/MS analysis, applying a requirement for metabolite presence in at least 4 out of the 5 replicates of untreated sample (0 min) coupled with an identification score over 70 arising from a NIST 11 (National Institute of Standards and Technology, USA) mass spectral library search as the threshold, led to identification of a total of 53 metabolites. The majority of these metabolites (48 out of 53) are primary metabolites, as is expected for GC-MS analysis^33–35^, and fall into the categories of carbohydrates, carboxylic acids, fatty acids and lipids (Fig. 2 and Supplemental Table 1). For our LC-MS/MS analysis, the same requirement for presence in at least 4 out of the 5 replicates of untreated sample (0 min), coupled with an identification score threshold of ≥0.6 in MassBank, led to an identification of a larger number of metabolites (Fig. 2 and Supplemental Table 1). Under positive ion mode, 224 metabolites were identified, with nearly 80% involved in secondary metabolism. In particular, a number of carotenoids (subgroup of terpenoids) and flavonoids (subgroup of phenolics) were detected in positive mode (Fig. 2A and Supplemental Table 1). Under negative ion mode, 168 metabolites were identified, of which nearly two thirds were secondary metabolites, with the dominant group being phenolics (58 out of 168), followed by sugar nucleosides/ nucleotides, carbohydrates and carboxylic acids (Fig. 2A and Supplemental Table 1).

In total, 390 non-redundant metabolites were identified by our criteria in untreated *B. napus* guard cells. Each of the three datasets from GC-MS/MS and LC-MS/MS positive and negative ion modes contain a unique subset of metabolites and thus these methods are complementary (Fig. 2B and Supplemental Table 1). Only three metabolites, phenylalanine, ferulic acid, and sinapic acid, were identified by all three acquisition methods (Fig. 2B). Only 13 metabolites were found in common between GC-MS/MS and LC-MS/MS, while 42 were identified by both positive ion mode and negative ion mode in LC-MS/MS (Fig. 2B).

The KEGG (Kyoto Encyclopedia of Genes and Genomes) PATHWAY database (http://www.genome.jp/kegg/pathway.html) contains a collection of manually compiled KEGG pathway maps representing molecular interaction and reaction networks of metabolism and other functions, derived from multiple organisms, including *A. thaliana*, *B. napus*, and *B. rapa*, a parental ancestor of *B. napus*. Of the metabolites we identified, there were 286 metabolites with a KEGG compound index. Of these, 124 metabolites mapped to the metabolic pathways available in KEGG from the reference species *B. napus*. The same set of metabolites also mapped to the metabolic pathways of *B. rapa* in KEGG. Distribution of identified metabolites on the metabolic map indicates that a wide variety of metabolic pathways are reseprented in the *B. napus* guard cell metabolome (Supplemental Fig. S1 and Supplemental Table 1). There are several reasons for incomplete mapping of all of our identified metabolites: 1) not all metabolites are indexed in KEGG. For example, a few of the flavonoids identified in our profiling, especially those in the glycosidic form, are not available in KEGG (Supplemental Table 1), which is consistent with previous observations on other plant metabolomes^36^. 2) KEGG pathway maps are based on experimental knowledge of metabolism, which can be far from complete.

The Plant Metabolic Network (PMN, http://www.plantcyc.org/) is another valuable compendium of plant compound information^37^. Of the metabolites we identified, 128 are included in the *B. rapa* compound list from PMN, which contains over 1700 non-redundant metabolites. For the 262 metabolites without a hit in the PMN *B. rapa* compound list, 92 have one or more chemical derivatives in the PMN list (Supplemental Table 1). Together with those metabolites mapped to KEGG pathways, 174 metabolites known to be present in *B. rapa* and/or *B. napus* are present in our metabolome profiling, suggesting considerable metabolite diversity in *B. napus* guard cells. Additionally, 29 metabolites out of the 390 metabolites we identified in *B. napus* guard cells were also detected in a previous, targeted study on *A. thaliana* guard cells that quantified a set of 85 specific metabolites^12^ (Supplemental Table 1). Misra *et al*.^25^ and Geng *et al*.^26^ recently reported 268 and 358 metabolites, respectively, in the metabolite profiling of guard cell enriched epidermal peels from *B. napus*, in studies of metabolome responses to bicarbonate and elevated CO_2_, respectively. Our identified metabolome had an overlap of 62 and 74 metabolites with those datasets, respectively (Supplemental Table 1). Our metabolome profile (390 metabolites; 225 of which were not previously identified in other guard cell metabolome profiling datasets^12,25,26^) thus significantly expands our knowledge regarding the metabolome of this specialized cell type.

### Identification of ABA-responsive metabolic signatures in *B. napus* GCPs

**Figure 3:**
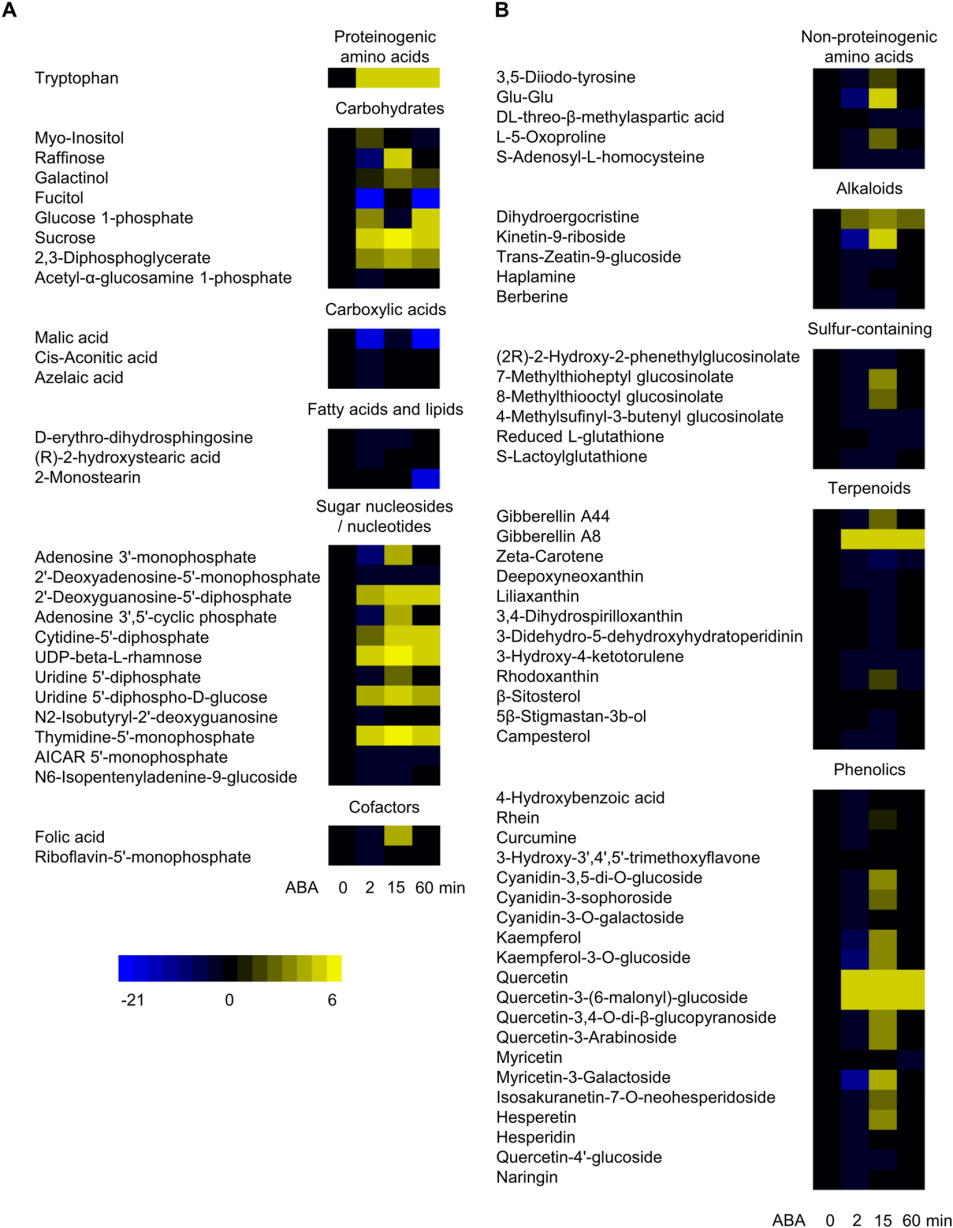
Primary (**A**) and secondary (**B**) metabolites responsive to ABA at different time points in *B. napus* GCPs. At 2, 15, and 60 min heat maps represent log2 of fold change, i.e., the log2-transformed metabolite abundance (peak area) at each time point divided by the level at 0 min; a 0 min column is also provided for comparison. All metabolites depicted were significantly changed at one or more time points (2 min, 15 min, and 60 min) of ABA treatment. Abbreviations: UDP: uridine diphosphate; AICAR: 5-aminoimidazole-4-carboxamide-1-ribofuranosyl.

*B. napus* GCPs were treated with ABA at a final concentration of 10 µM for 0 (i.e., untreated GCPs; results discussed above), 2, 15, or 60 min, respectively. Given that ethanol (EtOH, solvent control) did not regulate stomatal movement in *B. napus* (Fig. 1A) and no significant changes in metabolites caused by ethanol were detected in *A. thaliana* guard cells^12^, only GCPs treated with ethanol for 15 min were prepared in our experiment. Five replicates of each sample, i.e., 0 min, ABA 2 min, ABA 15 min, ABA 60 min, and EtOH 15 min, were prepared and analyzed on GC-MS/MS and LC-MS/MS in parallel. Principal component analysis (PCA) revealed that the 0 min and EtOH 15 min groups cluster together, whereas ABA treated groups are distinguished from 0 min and EtOH 15 min, indicating the ABA treatment as the major factor contributing to the cluster segregation (Supplemental Fig. S2). To identify responsive metabolites, we imposed a threshold of *p* value ≤0.05 in Student’s *t* test and at least 20% in fold change^38,39^. For these analyses, each treatment sample, i.e., ABA 2 min, ABA 15 min, ABA 60 min, and EtOH 15 min was compared to the 0 min sample. EtOH treatment for 15 min caused changes in only 12 metabolites, with 10, 1, and 1 identified from GC-MS/MS, LC-MS/MS positive mode, and LC-MS/MS negative mode, respectively (Supplemental Table 2). Eleven of these metabolites, for example, xylitol and palatinose, were also found in our ABA responsive dataset; these metabolites were not designated as ABA-regulated, due to their EtOH responsiveness. After combining the GC dataset with the two LC datasets, 17, 66, and 18 metabolites were found to be ABA responsive at time points 2 min, 15 min, and 60 min respectively, as compared to 0 min (Supplemental Table 2). The abundance changes of all these metabolites (77 unique metabolites in total) at different time points under ABA treatment are represented by heat maps (Fig. 3). None of these 77 metabolites was absent (i.e., no detection) in untreated (0 min) samples but present in ABA-treated samples, although some unidentified MS peaks appeared upon ABA treatment. Among the ABA responsive metabolites, 8, 27, and 48 metabolites were revealed by GC, LC positive mode, and LC negative mode, respectively. There was only one metabolite (galactinol) common to GC and LC negative mode, no metabolites common to GC and LC positive mode and only 5 metabolites common to LC positive mode and negative mode (5-aminoimidazole-4-carboxamide-1-ribofuranosyl 5′-monophosphate, 2,3-diphosphoglycerate, 2′-deoxyadenosine-5′-monophosphate, S-lactoylglutathione, and kaempferol) (Supplemental Table 2), again illustrating the value of multiple analysis methods. The major groups of the ABA responsive metabolites were phenolics (mostly flavonoids), carbohydrates, terpenoids (mostly tetraterpenoids), sugar nucleosides/nucleotides, and sulfur-containing metabolites (Fig. 3 and Supplemental Table 2).

**Figure 4:**
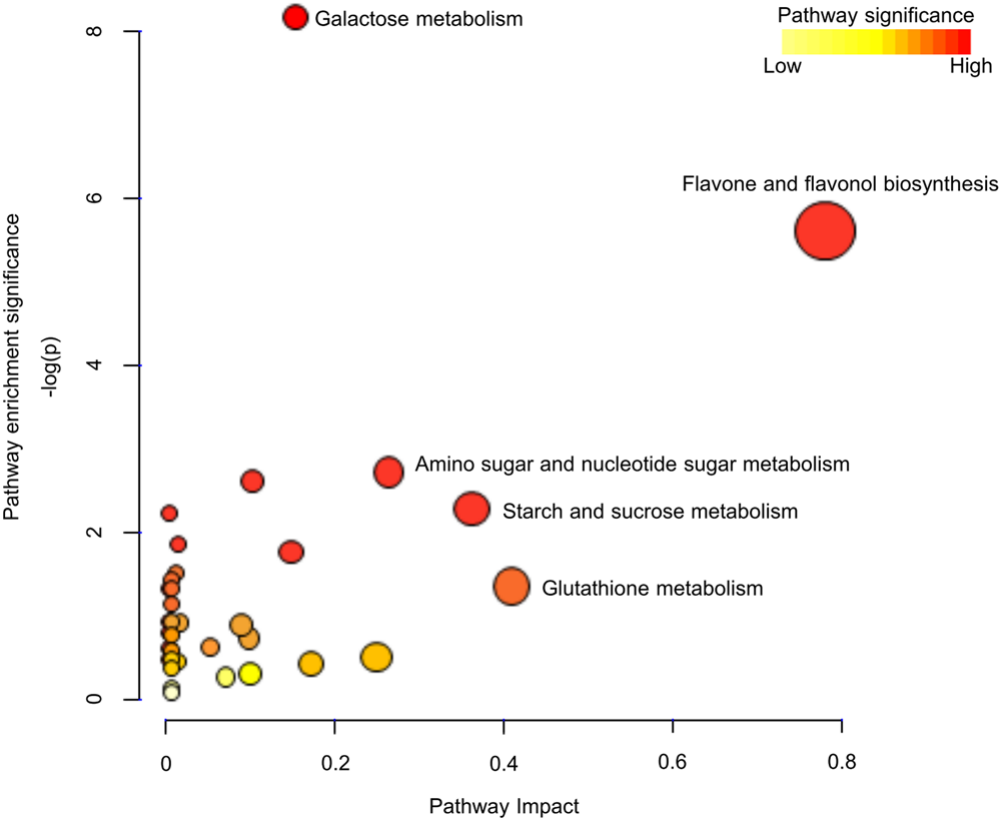
Metabolic pathways affected by ABA treatment in guard cells revealed by pathway analysis. x axis represents the impact of the identified metabolites on the indicated pathway. y axis indicates the extent to which the designated pathway is enriched in the identified metabolites. Values were ascertained from MetaboAnalyst. Circle colors (see color scale for reference) indicate pathway enrichment significance. Circle size indicates pathway impact.

**Figure 5:**
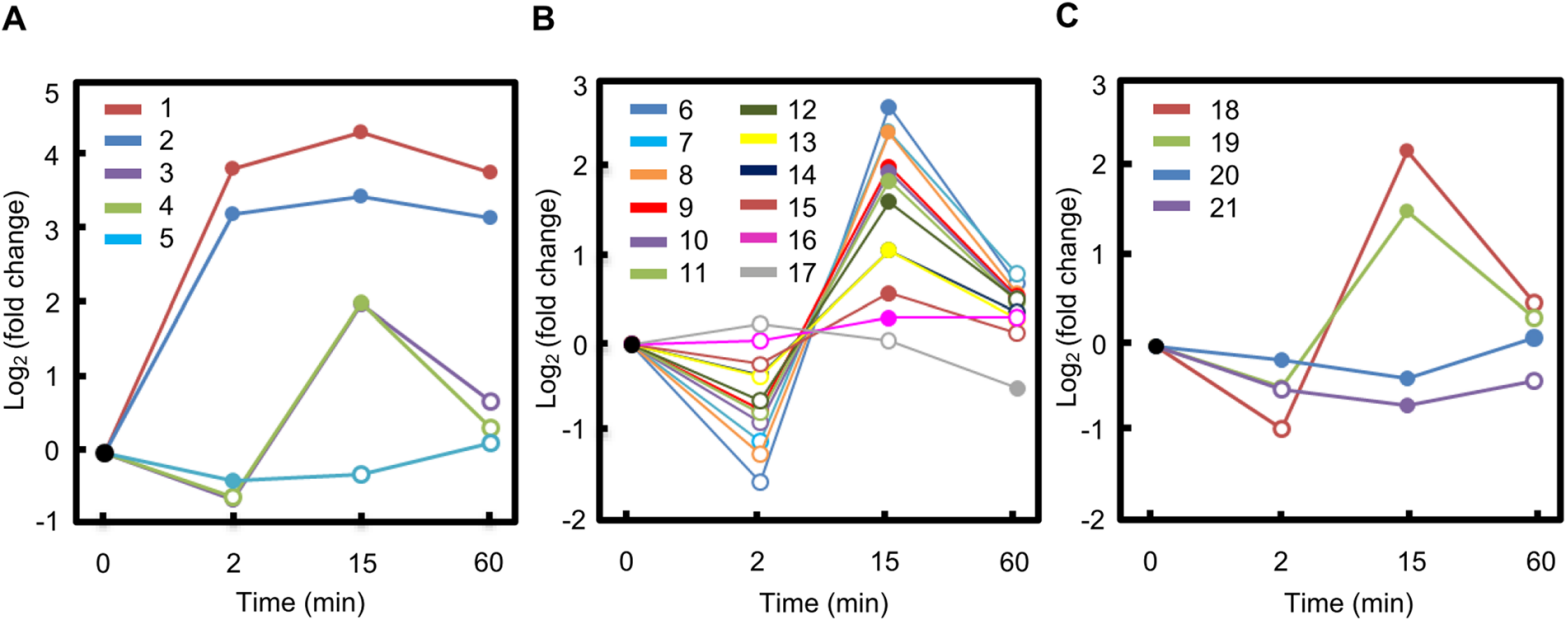
Abundance changes along the time course of ABA treatment for quercetin and quercetin derivatives (**A**), non-quercetin flavonoids (**B**), and glucosinolates (**C**). Metabolites 1–22 are: 1: quercetin-3-(6″-malonyl)-glucoside; 2: quercetin; 3: quercetin-3-arabinoside; 4: quercetin-3,4′-O-di-beta-glucopyranoside; 5: quercetin-4′-glucoside; 6: myricetin-3-galactoside; 7: kaempferol; 8: kaempferol-3-O-glucoside; 9: cyanidin-3,5-di-O-glucoside; 10: hesperetin; 11: isosakuranetin-7-O-neohesperidoside; 12: cyanidin-3-sophoroside; 13: hesperidin; 14: naringin; 15: cyanidin-3-O-galactoside; 16: 3-hydroxy-3′,4′,5′-trimethoxyflavone; 17: myricetin; 18: 7-methylthioheptyl glucosinolate; 19: 8-methylthiooctyl glucosinolate; 20: (2 R)−2-hydroxy-2-phenethylglucosinolate; 21: 4-methylsufinyl-3-butenyl glucosinolate. Solid data points indicate statistically significant changes upon ABA treatment (Student’s *t* test; *p* value < 0.05) compared to 0 min data.

A pathway enrichment analysis was performed using all available KEGG IDs of the ABA responsive metabolites (58 out of 77) against the KEGG *A. thaliana* reference metabolome using MetaboAnalyst 3.0^40^. Figure 4 shows all identified pathways from pathway enrichment analysis, which assesses the over-representation of inquiry compounds in known pathways, and their pathway impact values from pathway topology analysis, which indicate the importance of the identified metabolites to that pathway^41^ (Supplemental Table 3). Enriched pathways with high impact include flavone and flavonol biosynthesis, amino sugar and nucleotide sugar metabolism, and starch and sucrose metabolism (Fig. 4). Flavone and flavonol are two subgroups of flavonoids that are widely distributed secondary metabolites in higher plants^42^. The majority (15 out of 17) of the flavonoids were upregulated by ABA at 15 min (Fig. 5A and B; Supplemental Table 2). Sugar metabolism is also highly impacted by ABA treatment (Fig. 4). For example, an increase in sucrose and glucose 1-phosphate was observed in *B. napus* GCPs under ABA treatment (Fig. 3). Uridine 5′-diphosphate (UDP), UDP-glucose, and UDP-rhamnose also showed significant upregulation by ABA treatment (Fig. 3).

### Effects of flavonoids, glucosinolates, and sterols in stomatal responses to ABA in *A. thaliana* and *B. napus*

Multiple flavonoids were identified as ABA-responsive in *B. napus* guard cells (Supplemental Table 2, Fig. 3, and Fig. 5A and B), most of which were glycosylated, i.e., linked with a sugar moiety. Although such conjugated metabolites are generally presumed to be inactive^43^, one study found that flavonoid glycosides such as quercetin 3-O-glucoside and kaempferol 3-O-glucoside exhibit radical scavenging activities, a feature that would suppress ROS^42^, which are known to promote stomatal closure^44,45^. We observed that quercetin and quercetin-3-(6″-malonyl)-glucoside were strongly induced by ABA (Fig. 5A). Two other quercetin derivatives, quercetin-3,4′-O-di-beta-glucopyranoside and quercetin-3-arabinoside were also induced while quercetin-4′-glucoside was slightly repressed (Fig. 5A and Supplemental Table 2). All of the other 12 non-quercetin related flavonoids were significantly induced by ABA treatment at 15 min except for myricetin (Fig. 5B and Supplemental Table 2).

**Figure 6:**
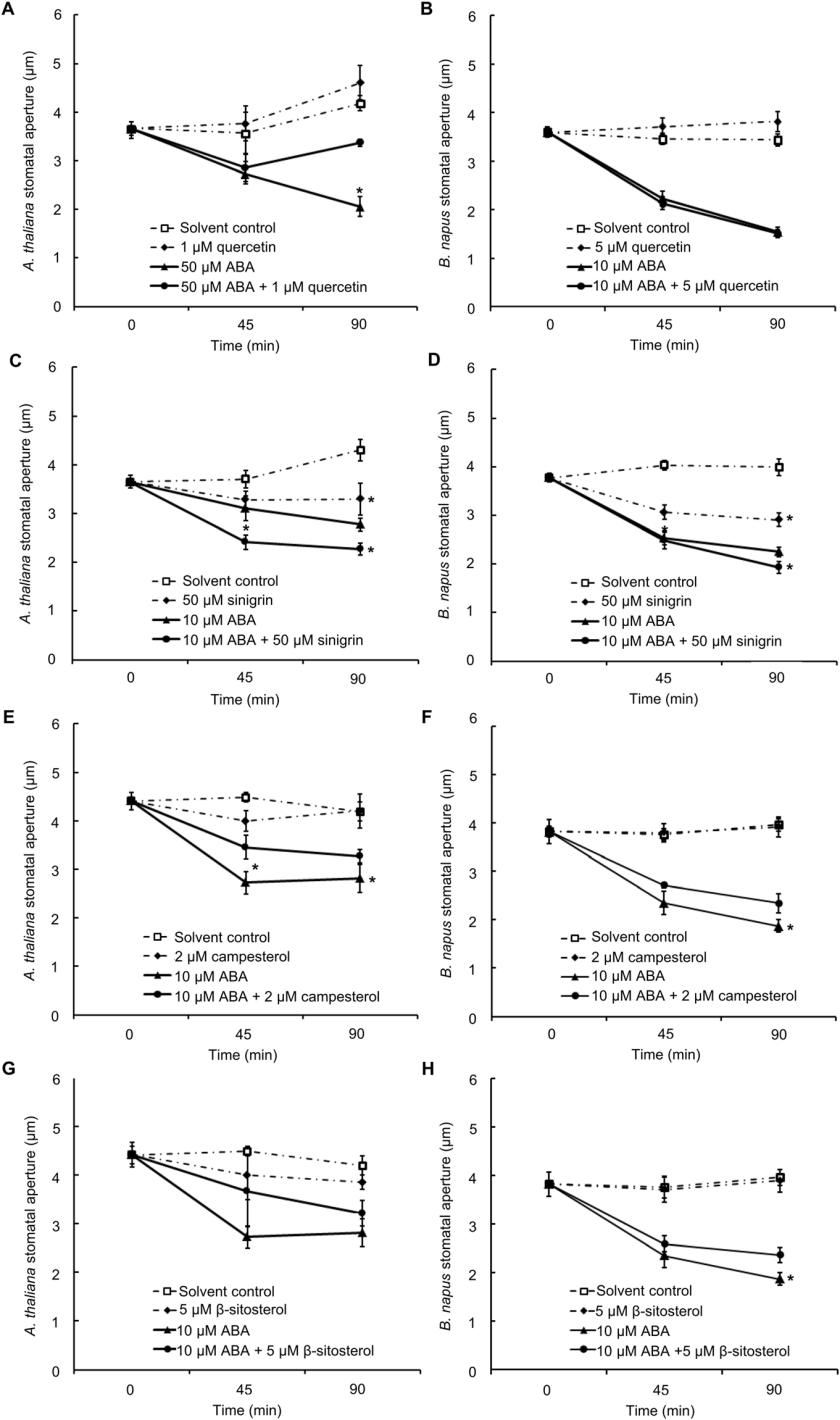
Effects of quercetin, sinigrin, campesterol, and β-sitosterol on stomatal apertures in *A. thaliana* (**A**,**C**,**E** and **G**) and *B. napus* (**B**,**D**,**F** and **H**) leaves. Data are means ± standard errors of at least 4 independent replicates with 100 ± 5 stomata measured for each sample. Asterisks indicate a significant effect of addition of the secondary metabolite (Student’s *t* test; *p* < 0.05).

Based on the results of our large-scale metabolite analyses and previous observations^46,47^, we hypothesized that the identified flavonoids would modulate stomatal movements. As a test of this hypothesis, we applied a non-glycosidic form of one of the strongly ABA-upregulated flavonoids, quercetin, to investigate its effect on stomatal movement and its regulation by ABA in *A. thaliana*. Based on quercetin measurement in the leaves of *A. thaliana* ^47,48^, we estimated that *in vivo* quercetin concentration is close to 1 µM. In Arabidopsis leaves, application of 1 µM quercetin caused a slight increase in stomatal aperture compared to solvent control after 90 min treatment; however, without statistical significance (Fig. 6A). On the other hand, ABA-induced stomatal closure was opposed by 1 µM quercetin (Fig. 6A), suggesting an antagonistic role of quercetin in the ABA signaling pathway. A significant effect of quercetin was observed at 90 min, implying the interaction of quercetin with ABA either as a late stage signaling event or a sustained process. We also tested the effect of quercetin in *B. napus* leaf pieces. However, an antagonistic effect of quercetin in ABA (10 µM) -induced stomatal closure was not observed in *B. napus* even at concentrations up to 5 µM (Fig. 6B). The inconsistency of quercetin effect between the two species might be caused by species-dependent sensitivity to the metabolite tested.

Several glucosinolates were also found to be responsive to ABA in our guard cell metabolomes, with 7-methylthioheptyl glucosinolate and 8-methylthiooctyl glucosinolate significantly increasing after ABA treatment (Fig. 5C and Supplemental Table 2). The glucosinolate-myrosinase system is a defensive mechanism uniquely present in some plant families, including the *Brassicaceae* ^49^. Stomatal movement modulation by components in this system also has been recognized^50–52^. To investigate glucosinolate regulation of stomatal movement, we applied an allyl-glucosinolate, sinigrin, separately or with ABA, to *A. thaliana* and *B. napus* leaves. Sinigrin is a naturally occurring metabolite in *A. thaliana* and *B. napus*, and is hydrolyzed by myrosinases into allyl isothiocyanate and allyl cyanide^53,54^. Sinigrin-induced stomatal closure and an additive effect with 10 µM ABA in promotion of stomatal closure were observed with statistical significance in both *A. thaliana* (Fig. 6C) and *B. napus* (Fig. 6D).

Several phytosterols (β-sitosterol, 5β-stigmastan-3b-ol, and campesterol) were downregulated in guard cells upon ABA treatment (Fig. 3 and Supplemental Table 2). We applied campesterol (2 µM), either separately or with ABA (10 µM), to *A. thaliana* and *B. napus*. In both species, an antagonistic effect of campesterol on ABA-induced stomatal closure was observed (Fig. 6E and F). The effect of another phytosterol, β-sitosterol was also tested in the ABA-induced stomatal closure of *A. thaliana* and *B. napus*. An antagonistic effect of β-sitosterol (5 µM) in ABA- induced stomatal closure was observed in *B. napus* (Fig. 6H) but not consistently in *A. thaliana* (Fig. 6G).

## Discussion

### Complementary GC-MS and LC-MS platforms together with simplified extraction enhances coverage in metabolome profiling

Largely due to the differences in the ionization techniques and columns for molecule separation, GC-MS and LC-MS each exhibit detection biases for certain classes of metabolites. Temperature gradients for separation and electron ionization are commonly used in GC-MS, and primary metabolites, such as amino acids, carbohydrates, organic acids, and fatty acids are the main categories of metabolites detected with GC-MS^33^. LC-MS usually separates molecules based on their polarity and uses soft electrospray ionization, which in practice covers a wider range of metabolites, including plant secondary metabolite groups such as alkaloids, phenolics, and flavonoids^33^. These platform biases were also observed in our study: the majority of metabolites identified with GC-MS were primary metabolites whereas LC-MS analyses detected both primary and secondary metabolites (Fig. 2 and Supplemental Table 1). The three analysis conditions are complementary and overall 390 non-redundant metabolites were identified, making this dataset one of the largest to date for a plant single cell type^22,55^.

Ionization in LC-MS can generally be divided into positive mode, in which samples are protonated, and negative mode, in which samples are deprotonated. Typically, certain categories of metabolites can be preferentially resolved under a certain mode. For example, in our experiments, carotenoids were only detected under positive mode (Supplemental Table 1), mostly with the MS precursor in the radical cation form [M]*^+^ rather than the protonated form [M + H]^+^, a phenomenon that has been observed before^56^. On the other hand, more acids were identified under negative mode, which might be due to the prone-to-deprotonate feature of acids (Supplemental Table 1). Therefore, as previously known and as we observed in our study, no single analytical instrument is entirely robust to cover the whole metabolome profile. For broad coverage, it is necessary to utilize multiple platforms for metabolome profiling.

In addition to the choice of the instrumentation platforms, the selection of the metabolite extraction protocol is another key factor that influences metabolome coverage. Practically, only a fraction of the entire metabolome can be resolved, in part depending on the composition of the extraction solution. For example, inclusion of chloroform in the extraction solvent was found to be counterproductive in an untargeted LC-MS metabolomics workflow^33^. Additionally, metabolites, even if successfully extracted, might not be detected by mass spectrometry due to failure to be dissolved by the loading buffer prior to the analysis. Therefore, an unbiased and efficient extraction protocol is critically important to successful metabolome profiling. Although responsive to stimuli, GCPs lack cell walls, and so can be easily disrupted for metabolite release. Accordingly, in this study metabolites were extracted from GCPs simultaneously with derivatization, including heating and shaking steps for GC-MS/MS analysis, and vortexing with loading buffer for LC-MS/MS analysis. This procedure dramatically reduced sample processing time and potential sample loss caused by transfers during extraction.

### Metabolite identification and annotation

In the early days of mass spectrometry based metabolomics, *m/z* was used for identification, sometimes together with chromatographic retention time. A major disadvantage of this type of identification is that isomers and stereoisomers cannot be differentiated due to their identical mass and occasional co-elution on chromatography^57^. With the advent of tandem mass spectrometry, fragmentation patterns (MS/MS or MS^n^) of a compound provide another important, and often defining, feature for metabolite identification^58^. Databases with mass spectral information are essential references for metabolomics studies. MassBank is the first public database of metabolite mass spectra, and over 50% are MS/MS or MS^n^ spectra. These spectra were acquired on a variety of instruments (~30 types) under independent conditions and contributed by ~30 research groups worldwide. In MassBank, the function “Spectrum Search” retrieves spectra that are similar to the experimentally-acquired inquiry spectrum, and a similarity score (within the range 0–1) is calculated to indicate the confidence of identification. MassBank shows superior performance for metabolite identification using tandem mass spectral search^59,60^. Due to its public availability, large number of reference spectra, and user-friendly scoring system, we chose MassBank to annotate the features acquired from our LC-MS/MS analysis. Using 0.6 as the threshold for similarity score^61^, we identified 224 non-redundant compounds from 2620 monoisotopic features acquired under positive mode and 168 non-redundant compounds from 2327 monoisotopic features acquired under negative mode. Together with the data from our GC-MS/MS analysis, identification of these metabolites greatly improves our knowledge of the guard cell metabolome.

### *B. napus* guard cell metabolome is related to stomatal function

Unlike the guard cell transcriptome^8,11^ and proteome^10,14^, the guard cell metabolome has remained largely unknown, in part due to the greater difficulty of sample preparation and technical limitations in metabolomics^18^. Here we employed complementary non-targeted metabolomics platforms for guard cell metabolome profiling and identified metabolites of both primary and secondary metabolism.

Primary metabolites are central components of the *B. napus* guard cell metabolome (Supplemental Table 1 and Fig. 2). Previous studies suggested that guard cells contain fewer chloroplasts and possess lower photosynthetic capability than mesophyll cells^62^. Here we identified a variety of sugars including monosaccharides (fructose and glucose), disaccharides (sucrose), and polysaccharides in *B. napus* guard cells (Supplemental Table 1). These carbohydrates might have been either previously synthesized elsewhere and transported into guard cells or generated by guard cell photosynthesis^62^. Guard cells contain abundant mitochondria and exhibit high rates of mitochondrial respiration^63,64^ and energy related proteins were also found to be enriched in *B. napus* guard cells^17^. The carboxylic acids and fatty acids found in our metabolite analysis are potential carbon sources to fuel stomatal movement, consistent with a recent report on the importance of triacylglycerol metabolism to blue light-induced stomatal opening^65^.

Plant secondary metabolites are usually low in abundance but with important functions not only for plants but also for human nutrition and medicine^66^. Major groups of plant secondary metabolites include phenolics, terpenoids, and nitrogen-containing metabolites (such alkaloids and amines). Flavonoids are a large family of plant secondary metabolites with benzo-γ-pyrone structure^67^. We identified a total of 76 flavonoids in *B. napus* guard cells (Supplemental Table 1). Flavonoids have antioxidant activity and ROS, e.g., H_2_O_2_ and reactive nitrogen species (RNS), e.g., NO, not only serve as secondary messengers in guard cell signal transduction but also cause mild oxidative stress in guard cells^14,44^. It can be speculated that flavonoids might participate in redox homeostasis maintenance for proper responsiveness of the guard cell system^47,68,69^.

Glucosinolates are hydrophilic nitrogen and sulphur containing glycosides that participate in responses to abiotic and biotic stresses^49,70^. At least 11 different glucosinolates have been identified and quantified in *B. napus* leaves^71^. We identified 7 aliphatic and 2 aromatic glucosinolates in *B. napus* guard cells (Supplemental Table 1), some of which were previously detected in other organs of *B. napus* or related species. For example, glucoerucin was detected in *B. napus* hypocotyl and cotyledon but not in seeds^72^; 3-methylthiopropyl and 3-methylsulfinylpropyl glucosinolates were detected in *B. oleracea* seeds^73^.

Plant sterols and steroid hormones are essential for plant development, reproduction, and responses to various abiotic and biotic stresses, including drought, salt, heat, cold, hypoxia, pesticides, and heavy metals^74^. Several *A. thaliana* brassinosteroids (BR)-related mutants are affected in stomatal function^75,76^ and a possible interaction between BR and ABA has been suggested^77^. *B. napus* guard cells are rich in steroids (Supplemental Table 1), suggesting a possible correlation to stomatal function in stress responses.

A recent study observed that ~45% stomata of a guard cell specific chlorophyll-deficient mutant were continuously closed, suggesting that photosynthesis is critical to maintain turgor in guard cells^78^. Additionally, the guard cell chloroplast is a site for light and CO_2_ sensing in guard cells^62,79,80^. Identification of tetraterpenoids, including several carotenoids, provides metabolite details for guard cell chloroplasts (Supplemental Table 1). For example, violaxanthin is a key component in both carotenoid biosynthesis and the xanthophyll cycle, and has been detected previously in *Vicia faba* guard cells^81,82^. Taken as a whole, the metabolome profiling revealed by non-targeted metabolomics is a valuable resource to improve our understanding of guard cell function.

### ABA responsive metabolites revealed by non-targeted metabolomics reveal new insights on ABA signaling in guard cells

Targeted and non-targeted metabolomics are complementary discovery approaches. The targeted strategy, analogous to reverse genetics, is powerful for hypothesis driven studies with an *a priori* list of metabolites of interest, and is especially useful for low abundance metabolites such as phytohormones. The disadvantage of targeted metabolomics is that it will not provide a global view of the response and will miss metabolites that were not previously hypothesized to be of interest. Non-targeted metabolomics, analogous to forward genetics, does not suffer from these shortcomings.

There are both similarities and differences between the results of our present non-targeted study on *B. napus* and our previous targeted metabolomics study on ABA treated *A. thaliana* guard cells^12^. Decreases in malic acid and zeatin glucoside, as well as an increase in tryptophan were observed in ABA treated *B. napus* guard cells (Fig. 3 and Supplementary Table 2). This is consistent with our previous targeted metabolomics study on the ABA-regulated metabolome of *A. thaliana* guard cells^12^. Up-regulation of gibberellins (A8 and A44) and quercetin was found in our study and similar trends of gibberellins (A3 and A4) and quercetin were detected by Jin *et al*.^12^ but without statistical significance. One difference between the two studies is that an increase of sucrose was observed in *B. napus* guard cells but a decrease in sucrose was observed in *A. thaliana* guard cells following ABA application (Fig. 3)^12^. These differences may have arisen from differences in species and/or ABA concentrations used for treatment.

Hormone crosstalk during ABA signaling has been observed in physiological, transcriptional, and targeted metabolomics studies^12,83^. In our study only a few phytohormones were identified, such as jasmonic acid, zeatin, and gibberellins (Supplemental Table 1). Other hormones such as auxin and ABA precursors, however, were not detected.

In our study, 77 metabolites were identified to be ABA responsive in *B. napus* guard cells, including 29 primary metabolites and 48 secondary metabolites. A majority of them (67 out of 77) showed significant changes at 15 min, while fewer showed significant changes at the beginning (17 at 2 min) or the end (18 at 60 min) of treatment. Indeed, PCA analysis suggests that the ABA-regulated metabolome at 60 min may be returning to the baseline 0 min state (Supplemental Fig. S2). These dynamics suggest that ABA causes temporal and in some cases transient changes in metabolite abundance in *B. napus* guard cells, as was also observed in the targeted metabolomics analysis on the ABA-regulated guard cell metabolome in *A. thaliana* ^12^.

ABA signaling pathways in guard cells have been intensively studied due to ABA’s relevant roles in plant stress responses^3,4,7^. The role of reactive oxygen species (ROS) as secondary messengers in ABA signal transduction has been well documented^3,44,45^. ROS bursts also cause oxidative stress and change cellular redox homeostasis. Small molecules such as ascorbic acid and glutathione participate in the regulation of ROS homeostasis in plants^84^. A decrease in glutathione in response to ABA was observed in both *B. napus* guard cells (Fig. 3) and *A. thaliana* guard cells^12^. Abundance increases in protein components of ROS scavenging, e.g., ascorbate peroxidase and glutathione peroxidase as well as redox status of cysteines in a variety of proteins were observed in ABA-treated *B. napus* guard cell protoplasts in previous proteomic studies^10,14^. Taken together, these metabolomics and proteomics results add to results from genetic and pharmacological approaches^44,85^ that implicate redox homeostasis as an important regulatory mechanism associated with ABA signaling in guard cells.

In plants, flavonoid accumulation has been observed to be associated with oxidative stresses induced by environmental stimuli^67^. Pathway enrichment analysis revealed that flavone and flavonol biosynthesis was highly impacted by ABA treatment in *B. napus* guard cells (Fig. 4). More specifically, we observed upregulation of 15 flavonoids upon ABA treatment (Figs. 3 and 5, and Supplemental Table 2). This is consistent with the observation that a flavonol synthase (AT5G08640), a chalcone-flavanone isomerase (AT5G05270), and a riboflavin biosynthesis protein (AT2G22450) were induced by ABA at the transcript level in *A. thaliana* guard cell-enriched epidermal peels^11^. Moreover, it was recently reported that an *A. thaliana* chalcone synthase null mutant *tt4-2*, which is defective in flavonol synthesis, is hypersensitive to ABA in ABA induced stomatal closure. The authors propose that flavonol accumulation in wild-type guard cells suppresses ROS elevation and moderates stomatal closure^47^. In another study, accumulation of anthocyanins was observed in Arabidopsis lines *pap1-D* with constitutive expression of *PAP1* (*Production of Anthocyanin Pigment1*) and these lines were more drought tolerant compared to wild type and the flavonoid-deficient mutant *tt4* ^46^. Higher levels of total flavonols and total anthocyanins were detected in lines with overexpression of both *PAP1* and *MYB12/PFG1* (*Production of Flavonol Glycosides 1*). These lines showed comparable drought tolerance to *pap1-D* but higher survival rate after re-watering^46^. These results suggested a link between flavonoid accumulation and drought/ABA responses. However, a direct effect of flavonoids on ABA-induced stomatal closure has not been studied yet. Here we observed that ABA-induced stomatal closure is reduced by 1 µM quercetin in Arabidopsis (Fig. 6A), suggesting an antagonistic role of quercetin in the ABA signaling pathway. Together with the observation that several flavonoids were induced by ABA (Figs 3 and 5), our metabolite analysis supports the hypothesis^47,69^ that flavonoid accumulation in guard cells might function in a negative feedback process to mitigate or ensure only transient ABA-induced ROS elevation.

Sugar metabolism was also highly impacted by ABA treatment (Fig. 4). A >100 fold increase of sucrose was observed at 15 min of ABA treatment and the upregulation was maintained along the time course. Given that our analyses were perform on isolated guard cell protoplasts, the detected sucrose must be endogenous to guard cells, and might originate from guard cell photosynthesis or from starch degradation. The transcripts of two sucrose synthases (*SUS1* and *SUS3*), two sucrose-phosphate synthases (*SPS1* and *SPS3F*), as well as an α-glucan, water dikinase required for starch degradation (*SEX1*) were all induced by ABA in *A. thaliana* guard cell enriched epidermal peels^11^. Based on a sucrose standard curve established on the same GC-MS as used for non-targeted analysis, the sucrose concentration is estimated to be 10–15 µM at 0 min and ~2 mM at 15 min of ABA treatment in *B. napus* guard cells, respectively. Such an increase will not dramatically contribute to osmolarity increase inside the cell. It has been reported that external application of sucrose and its catabolic products glucose and fructose are sensed within guard cells by hexokinases to stimulate stomatal closure in tomato^86^; those results, together with ours, suggest that sucrose may play a role in transduction of the ABA signal in guard cells.

Sucrose might also be a carbon source for energy production during stomatal closure, which has been shown to be an energy-requiring process^62,87^. An increase in glucose 1-phosphate was also observed in *B. napus* GCPs under ABA treatment (Fig. 3). The phosphorylated glucose could be catabolized for ATP production through glycolysis and/or tricarboxylic acid (TCA) cycle to energize stomatal closure^88^. Additionally, one TCA cycle product, malic acid (malate) was present at 0 min but was not detectable at the end of ABA treatment (Fig. 3; Supplemental Table 2), suggesting the catabolism or export of this osmoticum during stomatal closure^89^, e.g., through R-type and S-type anion channels, which are permeable to malate^90,91^.

Glucosinolates are nitro-sulphate secondary metabolites that are present in many *Brassicaceae* species, including *A. thaliana* and *B. napus*. Glucosinolates are degraded by myrosinases, and resulting hydrolysis products such as thiocyanates, isothiocyanates and nitriles are known to deter insects and pathogens^49^. Involvement of the glucosinolate-myrosinase system in stomatal movement regulated by abiotic stress was not recognized until a report of a myrosinase mutant, *tgg1*, exhibiting hyposensitivity to ABA inhibition of guard-cell inward K^+^ channels and stomatal opening^50^. A subsequent study suggested that *A. thaliana* myrosinases TGG1 and TGG2 function downstream of ROS production and upstream of cytosolic Ca^2+^ elevation during ABA and MeJA signaling in guard cells^51^. Methionine chain elongation of glucosinolates is catalyzed by isopropylmalate dehydrogenase (IPMDH) and *A. thaliana ipmdh* mutants exhibit hyposensitivity in both ABA promotion of stomatal closure and ABA inhibition of opening^14^. Additionally, stomatal closure induced by glucosinolate hydrolysis products such as isothiocyanate was also reported^52^.

Consistent with previous reports suggesting that glucosinolate hydrolysis products function analogously to ABA in stomatal aperture regulation, we observed sinigrin-induced stomatal closure that was additive with ABA in both *A. thaliana* and *B. napus* (Fig. 6B and D). One degradation product of sinigrin, allyl isothiocyanate (AITC), was found to induce stomatal closure in both *A. thaliana* and *V. faba* accompanied by ROS and NO production^52,92^. AITC-induced cytosolic Ca^2+^ oscillation was also observed in *A. thaliana* guard cells^52^. Therefore, the effect of sinigrin that we observe here might be mediated through stimulated production of these second messengers, which are known to promote stomatal closure.

Plant sterols (phytosterols) are structurally related to cholesterol with differences in side chains^93^. Phytosterols are membrane components with various biological functions, including regulation of membrane fluidity and permeability^94^. The most common natural phytosterols species include campesterol, stigmasterol, β-sitosterol, and brassicasterol^94^. Linkage between phytosterols and drought tolerance has been documented in several plant species^95^. For example, elevation in contents of sterols (campesterol, stigmasterol, and β-sitosterol) and steryl esters, along with enhanced enzyme activity of 3-hydroxy-3-methylglutaryl coenzyme A (HMG-CoA) reductase, a key enzyme of phytosterol biosynthesis, were observed in rice seedlings under drought stress^95^. Transgenic rice with the *SQUALENE SYNTHASE* (*SQS)* gene disrupted by RNAi had lower sterol and BR content, and showed enhanced drought tolerance^96^. In the present study, decreases in β-sitosterol, 5β-stigmastan-3b-ol, and campesterol were observed in ABA treated *B. napus* guard cells (Fig. 3 and Supplemental Table 2). These results seem contradictory to the observation in rice^95^. However, this might be due to the differences in material (GCPs vs. whole plant) and treatment duration (1 h vs. days). The decreases in sterol content we observed in GCPs might be caused by membrane recycling due to ABA-induced cellular shrinkage (Fig. 1B). External application of phytosterols, in general, had an antagonistic effect in ABA-induced stomatal closure (Fig. 6E, F, and H). Such exogenous phytosterols might contribute to maintenance of membrane stability under ABA treatment or retard ABA signal perception and transduction.

## Conclusions

Non-targeted metabolomics provides robust discovery workflows to reveal metabolome profiles and their roles in plant stress responses. Here we employed both GC-MS/MS and LC-MS/MS platforms and identified a total of 390 non-redundant metabolites in *B. napus* guard cells, which have not previously been subjected to metabolome profiling. Temporal metabolite changes upon ABA treatment were also investigated. An overall increase in flavonoids, divergent changes in glucosinolates, and a decrease in phytosterols upon ABA treatment were detected in *B. napus* guard cells. Involvement of quercetin, sinigrin, β-sitosterol, and campesterol in regulation of stomatal closure was confirmed in *A. thaliana* and/or *B. napus*, demonstrating the conserved nature of several of the metabolite-based regulatory mechanisms. ABA responsive metabolic signatures are potential targets for engineering enhanced drought tolerance for crop improvement.

## Materials and Methods

### Plant growth, guard cell protoplasting, and ABA treatment

*Brassica napus* double haploid line (DH12075) plants were grown under an 8-hour-day (22 °C) /16-hour-night (20 °C) cycle with light intensity 125 µmol·m^−2^·sec^−1^. Fully expanded leaves from 6-7 week old plants were used for guard cell isolation as previously described^17,19^. Briefly, ~30 g of fully expanded leaves with main veins excised were blended 4-5 times for 30 s each in cold tap water using a blender (Oster Inc., USA). After blending, epidermal peels were washed thoroughly with tap water and transferred to 200 mL of the first enzyme solution: 0.1% (w/v) PVP-40, 0.25% (w/v) BSA fraction V, 0.7% Cellulase R-10, and 0.02% Macerozyme R-10, prepared in 55% (v/v) basic solution. Basic solution contains 0.55 M sorbitol, 0.5 mM CaCl_2_, 0.5 mM MgCl_2_, 0.5 mM ascorbic acid, 10 µM KH_2_PO_4_, 10 mM MES-Tris, pH 5.5. Peels were digested for 1 h at 28-29 °C with a shaking speed of 140 rpm. Peels were then retained by filtration with 100 µm mesh and transferred to the second enzyme solution: 200 mL, 0.25% (w/v) BSA fraction V, 1.1% (w/v) Onozuka RS cellulase, and 0.02% (w/v) Pectolyase Y-23 prepared in 100% basic solution. Digestion was for ~1.5 h at 20 °C with a shaking speed of 50 rpm. Peels were then retained on 30 µm nylon mesh and were rinsed with 600–800 mL basic solution, resulting in a filtrate which contained the guard cell protoplasts. Protoplasts were collected by centrifugation at 150 *g* for 5 min and carefully layered on top of the same volume of Histopaque (−1077, Sigma-Aldrich Co., USA), followed by centrifugation at 150 *g* for 15 min. Intact GCPs retained between the two phases were collected using a transfer pipette and washed thoroughly with basic solution. The protoplasts were finally resuspended in 5 mL of basic solution. The yield of protoplasts was estimated with a hemocytometer (Hausser Scientific, USA).

Guard cell protoplasts were allowed to recover under room light (8 ± 1 µmol·m^−2^·sec^−1^) at room temperature (~21 °C) for 1 h, then aliquots (1 mL) of guard cell protoplasts (approximately 0.8–1.2 million protoplasts, varying among replicates) were treated with 1 µL ABA (10 mM stock, final concentration 10 µM ABA; A.G. Scientific Inc., USA) for 0 (no ABA), 2, 15, or 60 min, respectively. Guard cell protoplasts treated with ethanol (solvent control) for 15 min were also prepared. At the end of the treatment, guard cell protoplasts were collected by centrifugation at 150 *g* for 5 min at 4 °C. Each guard cell protoplast pellet was frozen with liquid nitrogen and stored at −80 °C for future analysis.

### Stomatal bioassays in *B. napus* and FDA staining for monitoring of protoplast diameter

Leaf pieces or abaxial epidermal peels were prepared from fully expanded leaves excised before onset of the light period in the growth chamber, and then incubated with opening solution (5 mM KCl, 0.1 mM CaCl_2_, 10 mM MES-KOH, pH 6.15) for 3 h under light (intensity of 150 ± 25 µmol m^−2^ s^−1^) to promote stomatal opening. ABA or the solvent control (ethanol) was then added to the solution at a final concentration of 10 µM or 0.1% (v/v) respectively. At the indicated time points, leaf pieces or epidermal peels were observed under light microscopy (Carl Zeiss Inc., USA). Stomatal apertures were measured by analysis of the digital images using ImageJ (National Institutes of Health, USA). Each experiment was repeated three times with 105 ± 5 stomata measured for each sample.

To measure protoplast diameters, guard cell protoplasting and ABA treatment were carried out as described above, then guard cell protoplasts were collected by centrifugation and FDA (final concentration 5 µM) was added for ~1 min to allow the dye to permeate through cell membranes. FDA was excited by the 488 nm line of the argon laser and detected using a bandpass emission filter (500–550 nm) and images were acquired using a laser scanning confocal microscope (LSM 510, Carl Zeiss Inc., USA). Diameters were measured by analysis of the digital images using ZEN (version 2012, Carl Zeiss Inc., USA). Each experiment was repeated three times with 105 ± 5 GCPs measured for each sample.

### Sample preparation and GC-MS/MS analysis

Before derivatization, pellets from 4–6 individual protoplastings were pooled to compose one biological replicate, which contained 4–4.5 million GCPs. Five replicates were prepared for GC-MS analysis. For GC-MS analysis, metabolite extraction and derivatization were conducted simultaneously through the derivation procedure. Briefly, 10 µL of methoxamine (MOX) reagent (Thermo Fisher Scientific Inc., USA) was added to each biological replicate and incubated at 28 °C for 90 min. Then 90 µL of N, O-bistrifluoroacetamide (BSTFA) + 1% trimethylchlorosilane (TMCS) (Thermo Fisher Scientific Inc., USA) was added to each sample, followed by shaking at 400 rpm at 60 °C for 1 h. After centrifugation for 15 min at 12000 rpm, the supernatant of each sample was transferred to a glass auto-sample vial. Samples were then injected in a randomized order, with 0.5 µL of each sample injected into an Agilent 7980 A/5975 C GC-MS (Agilent Technologies, USA) with a 37.5 min temperature gradient: 50 °C for 1 min then ramping to 315 °C at 10 °C/min followed by 315 °C for 10 min.

### Data analysis for GC-MS/MS datasets

For analysis of GC-MS data, peaks of each sample were detected and deconvoluted using Automated Mass Spectral Deconvolution and Identification System (AMDIS, National Institute of Standards and Technology, USA) with the following settings: Component width - 20; Adjacent peak subtraction - One; Resolution - High; Sensitivity - Medium; Shape requirement - Medium. Peak alignment across samples and feature identification were completed using Mass Profile Professional (MPP) software (Agilent Technologies, USA) with the following parameters: minimal ion number 4; mass tolerance 0.1 Da; retention time tolerance 0.3 min; appearance filter - 60% within one sample group. Spectra of all aligned peaks were searched against the NIST 11 mass spectral library (National Institute of Standards and Technology, USA) using the built-in ID Browser function in MPP. Metabolites with a score over 70 were considered as confident identification based on previous publications^97^. Peak areas were exported and normalized against input GCP number and were log2 transformed for statistical analysis. A *p* value ≤ 0.05 (Student’s *t* test) together with at least 20% change in abundance based on peak area were used as criteria to define metabolic features with significant changes between samples.

### Sample preparation and LC-MS/MS analysis

Before metabolite extraction, pellets from 4–6 individual protoplastings were pooled to compose one biological replicate, which contained 4–4.5 million GCPs. Five replicates were prepared for LC-MS/MS analysis. Metabolites were extracted by adding 50 µL of LC-MS injection solution (3% acetonitrile, 0.1% formic acid, with chlorpropamide as internal standard) and vortexed for 1 h at 4 °C. After centrifugation for 15 min at 12000 rpm, the supernatant of each sample was transferred to an auto-sample vial. Samples were randomized and analyzed on an HPLC-QTOFMS (Shimadzu Prominence UFLC XR and AB Sciex 5600 quadrupole time-of-flight mass spectrometry) platform. Five microliters of each sample was separated on an Acquity BEH C18 Column (100 × 2.1 mm 1.7 µm, Waters, USA) using a gradient with aqueous acetonitrile ramping from 3% to 90% in the mobile phase at a flow rate of 250 µL/min. Both positive and negative ion electrospray ionization mass spectra were acquired over the mass range 50–1250 Da. Data acquisition was under information dependent acquisition (IDA) mode with one 100 ms survey scan and up to twenty 100 ms MS/MS product ion scans per duty cycle.

### Data analysis for LC-MS/MS datasets

MS peaks from the LC-MS/MS datasets were extracted and aligned across all samples using MarkerView^TM^ software (AB Sciex Pte Ltd., USA) with the following parameters: retention times between 0 min and 20.00 min.; Subtraction Offset of 10 scans; Subtraction Mult. Factor of 1.3; Noise Threshold of 50; Min. Spectral Peak Width of 15 ppm; Min. RT Peak Width of 5 scans; Retention Time Tolerance of 0.25 min.; Mass Tolerance of 25.0 ppm; Maximum of 100,000 peaks^98^. These parameters were optimized using the Check Peak Alignment function of MarkerView^TM^.

For metabolome profiling (0 time point), presence of a MS peak in at least 4 out of 5 replicates was required for export of MS/MS data for metabolite identification. For ABA responsive feature identification, presence of a MS peak in at least 3 samples out of 25 samples (5 treatments with 5 replicates for each treatment) was first required, next, whether peak area was significantly changed by ABA treatment was assessed, and finally, for those showing significant changes, MS/MS data were exported for metabolite identification. Principal component analysis was performed with all detected features from the three analysis methods using MarkerView^TM^ with base-e logarithm of the peak areas as weighing option and Pareto as scaling option.

Peaks annotated as monoisotopic ions with an *m/z* between 100 and 800 and a retention time between 1.5 and 16 minutes were exported for further analysis. After normalization against the chlorpropamide peak area (internal standard), peak areas were log2 transformed for statistical analysis between sample groups. A *p* value ≤0.05 (Student’s *t* test) together with at least 20% change in normalized MS peak area were used as criteria to define metabolic features with significant changes between samples^38,39^. MS/MS spectra of peaks present in at least four replicates of 0 min (control) sample and those peaks showing significant changes upon ABA treatment were exported from PeakView^TM^ software (AB Sciex Pte Ltd., USA). Raw spectral intensity was converted to relative peak intensity ranging from 0–999 using Excel for each peak and submitted to MassBank (Nara Institute of Science and Technology, Japan) for identification. A similarity score for each hit is calculated by the MassBank search according to the method of Horai *et al*.^61^. We consider a hit with score greater than 0.6 as identified at identification level 2^61,99^; we report the metabolite with the highest score for each inquiry spectrum. Exogenous compounds such as drugs, herbicides, pesticides, and non-plant derived metabolites were manually excluded^99^. A general consensus has been reached for four levels of metabolite identification: 1) identified compounds, i.e., definitive identification; 2) putatively annotated compounds; 3) putatively characterized compound classes; and 4) unknown compounds^99,100^. It is proposed that definitive identification requires comparison of a minimum of two independent properties (e.g., retention time and mass spectrum, accurate mass and tandem MS) to an authentic compound standard analyzed on the same instrument and under identical conditions. Comparison to values reported for authentic compounds based on the literature or external laboratory data results in level 2 identifications^99^. The metabolites reported in our study meet the standards for this second level for identification.

### Metabolite mapping and pathway enrichment analysis

Of the 390 metabolites we identified, there were 286 metabolites with a KEGG compound index. The list of KEGG compound indices was submitted to KEGG for mapping against *B. napus* and *B. rapa* as reference organsims using the User Data Mapping function (http://www.genome.jp/kegg-bin/show_pathway?map01100). Mapped metabolites are indicated with black circles in the metabolic pathway overview (Supplemental Fig. S1) and are annotated as mapped in Supplemental Table 1. The compounds listed in PMN for *B. rapa* (Chinese cabbage) were downloaded from PMN (http://www.plantcyc.org/). The metabolites we identified were checked manually for presence in the PMN *B. rapa* compound list (Supplemental Table 1).

A pathway enrichment analysis was performed using all available KEGG IDs of the ABA responsive metabolites (a total of 58) against the KEGG *A. thaliana* reference metabolome (with 87 pathways) using MetaboAnalyst 3.0^40^. The hypergeometric test was used for over-representation analysis and relative-betweeness centrality was used for pathway topology analysis^39^. Results (Supplemental Table 3) were generated through MetaboAnalyst.

### Stomatal bioassays in *A. thaliana* and *B. napus*

The effects of quercetin, sinigrin, sitosterol, and campesterol were investigated in *A. thaliana* and *B. napus* grown under the same conditions as described above for *B. napus* GCP isolation. Fully expanded leaves from ~5 week old *A. thaliana* or ~6 week old *B. napus* leaf pieces (~5 mm × 5 mm) were excised before onset of the light period in the growth chamber, and then incubated with opening solution (5 mM KCl, 0.1 mM CaCl_2_, 10 mM MES-KOH, pH 6.15) for 2.5 h under white light (intensity of 150 ± 25 µmol m^−2^ s^−1^) to promote stomatal opening. Treatments were added as follows: ethanol as solvent control, 50 µM ABA with or without 1 µM quercetin (Sigma-Aldrich Co., USA), 10 µM ABA with or without 50 µM sinigrin (Sigma-Aldrich Co., USA), 10 µM ABA with or without 5 µM sitosterol (Avanti Polar Lipids, Inc., USA), or 10 µM ABA with or without 2 µM campesterol (Avanti Polar Lipids, Inc., USA). *B. napus* leaf pieces or *A. thaliana* abaxial epidermes peeled at indicated time points were used for stomatal image acquisition under a light microscope (Nikon Instruments Inc., USA) connected to a digital camera (Nikon Inc., USA). Stomatal apertures were measured by analysis of the digital images using ImageJ (National Institutes of Health, USA). Each experiment was repeated at least three times with 105 ± 5 stomata measured per sample. Treatments were blinded during image acquisition and analysis.

### Data Availability Statement

All data generated or analysed during this study are included in this published article (and its Supplementary Information files).

## Electronic supplementary material

Supplemental Figures

Supplemental Table 1

Supplemental Table 2

Supplemental Table 3
