# Supplementary material for: Metabolic Signatures in Response to Abscisic Acid (ABA) Treatment in Brassica napus Guard Cells Revealed by Metabolomics
Source: Sci Rep. 2017 Oct 9;7:12875. doi: 10.1038/s41598-017-13166-w (PMC5634414; doi:10.1038/s41598-017-13166-w)
Supplement: Supplementary file 1 — Supplemental Figures [file 41598_2017_13166_MOESM1_ESM.pdf]

Metabolic Signatures in Response to Abscisic Acid (ABA) Treatment in *Brassica napus* Guard Cells Revealed by Metabolomics

Mengmeng Zhu, Sarah M. Assmann

Supplementary information

Supplemental Figure S1. Mapping of identified metabolites from *B. napus* guard cells into KEGG metabolic pathways. Black dots represent mapped metabolites.

Supplemental Figure S2. Principal component analysis using all detected features from the combined GC-MS/MS and LC-MS/MS data sets.

Supplemental Table 1. Metabolites of *B. napus* guard cells identified from GC-MS/MS and LC-MS/MS platforms.

Supplemental Table 2. ABA-responsive metabolites in *B. napus* guard cell revealed by non-targeted metabolomics.

Supplemental Table 3. Pathway enrichment analysis report generated from MetaboAnalyst.

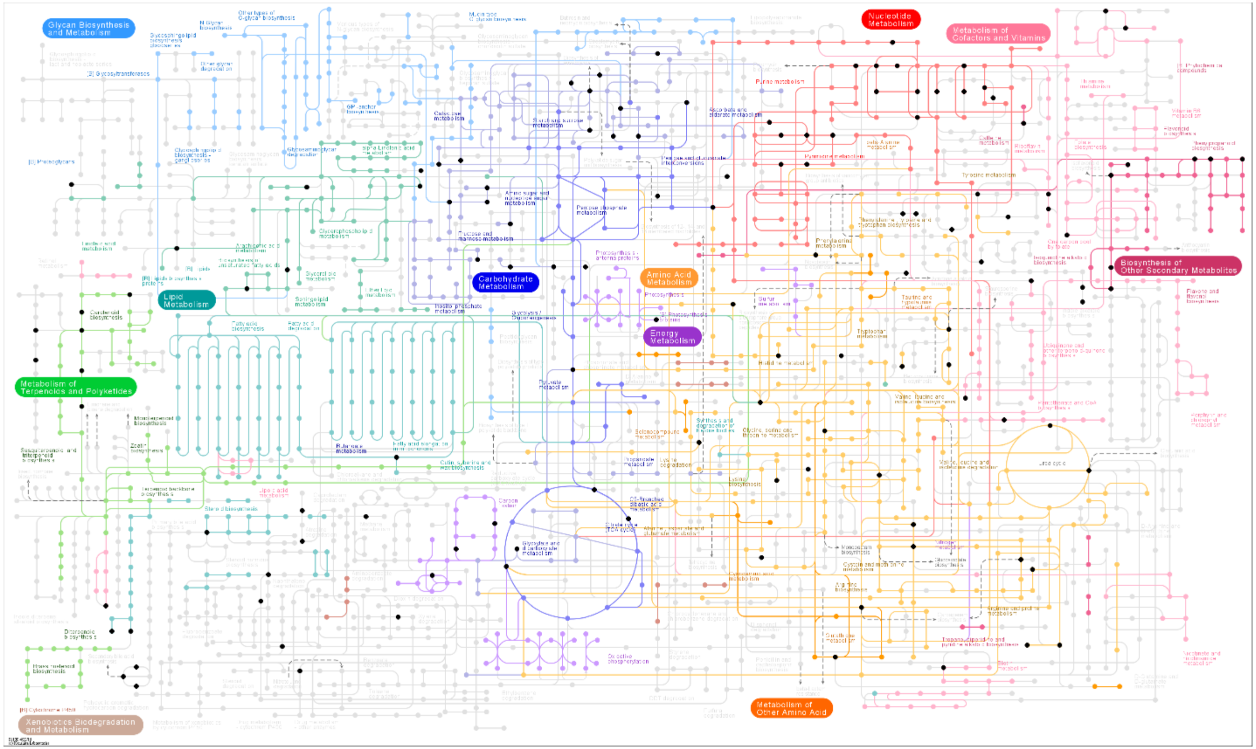

Supplemental Figure S1. Mapping of identified metabolites from *B. napus* guard cells into KEGG metabolic pathways. Black dots represent mapped metabolites.

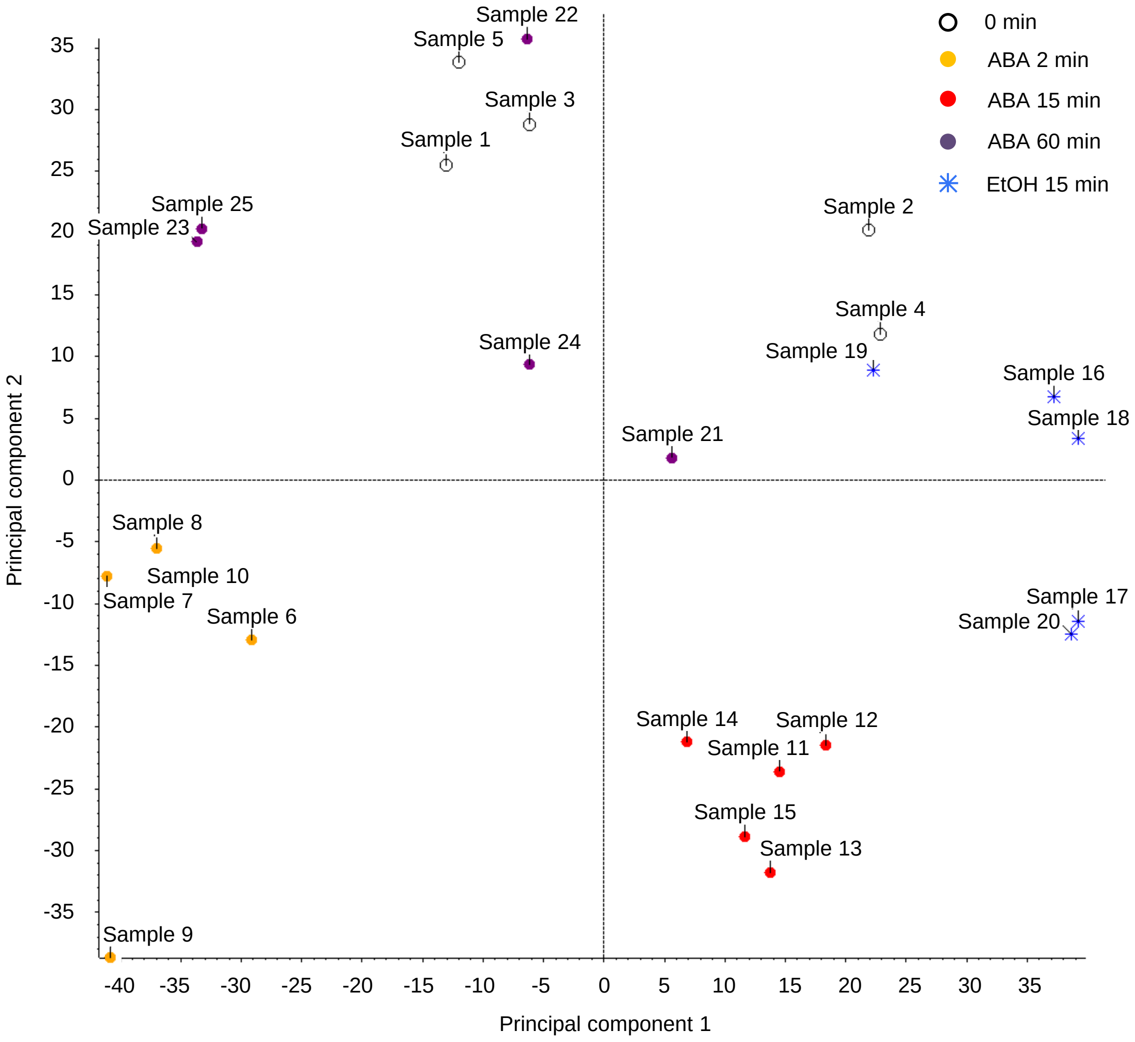

Supplemental Figure S2. Principal component analysis using all detected features from the combined GC-MS/MS and LC-MS/MS data sets.
